# Computer aided identification of a Hevein-like antimicrobial peptide of bell pepper leaves for biotechnological use

**DOI:** 10.1186/s12864-016-3332-8

**Published:** 2016-12-15

**Authors:** Patrícia Dias Games, Elói Quintas Gonçalves daSilva, Meire de Oliveira Barbosa, Hebréia Oliveira Almeida-Souza, Patrícia Pereira Fontes, Marcos Jorge deMagalhães-Jr, Paulo Roberto Gomes Pereira, Maura Vianna Prates, Gloria Regina Franco, Alessandra Faria-Campos, Sérgio Vale Aguiar Campos, Maria Cristina Baracat-Pereira

**Affiliations:** 1 Department of Biochemistry and Molecular Biology, Universidade Federal de Viçosa, Viçosa, MG 36570-900 Brazil; 2 Department of Plant Science, Universidade Federal de Viçosa, Viçosa, MG 36570-900 Brazil; 3 Embrapa Genetic Resources & Biotechnology, Brazilian Agricultural Research Corporation, Brasília, DF 70770-900 Brazil; 4 Department of Biochemistry and Immunology-ICB, Universidade Federal de Minas Gerais, Av. Antônio Carlos 6627, Belo Horizonte, MG 31270-901 Brazil; 5 Department of Computer Science-ICEX, Universidade Federal de Minas Gerais, Av. Antônio Carlos 6627, Belo Horizonte, MG 31270-901 Brazil

**Keywords:** Hevein-like, Antimicrobial peptide, Bell pepper, Plant defense, Peptidomics, Computational tools, Bioinformatics, Biotechnology

## Abstract

**Background:**

Antimicrobial peptides from plants present mechanisms of action that are different from those of conventional defense agents. They are under-explored but have a potential as commercial antimicrobials. Bell pepper leaves (‘Magali R’) are discarded after harvesting the fruit and are sources of bioactive peptides. This work reports the isolation by peptidomics tools, and the identification and partially characterization by computational tools of an antimicrobial peptide from bell pepper leaves, and evidences the usefulness of records and the in silico analysis for the study of plant peptides aiming biotechnological uses.

**Results:**

Aqueous extracts from leaves were enriched in peptide by salt fractionation and ultrafiltration. An antimicrobial peptide was isolated by tandem chromatographic procedures. Mass spectrometry, automated peptide sequencing and bioinformatics tools were used alternately for identification and partial characterization of the Hevein-like peptide, named HEV-CANN. The computational tools that assisted to the identification of the peptide included BlastP, PSI-Blast, ClustalOmega, PeptideCutter, and ProtParam; conventional protein databases (DB) as Mascot, Protein-DB, GenBank-DB, RefSeq, Swiss-Prot, and UniProtKB; specific for peptides DB as Amper, APD2, CAMP, LAMPs, and PhytAMP; other tools included in ExPASy for Proteomics; The Bioactive Peptide Databases, and The Pepper Genome Database. The HEV-CANN sequence presented 40 amino acid residues, 4258.8 Da, theoretical pI-value of 8.78, and four disulfide bonds. It was stable, and it has inhibited the growth of phytopathogenic bacteria and a fungus. HEV-CANN presented a chitin-binding domain in their sequence. There was a high identity and a positive alignment of HEV-CANN sequence in various databases, but there was not a complete identity, suggesting that HEV-CANN may be produced by ribosomal synthesis, which is in accordance with its constitutive nature.

**Conclusions:**

Computational tools for proteomics and databases are not adjusted for short sequences, which hampered HEV-CANN identification. The adjustment of statistical tests in large databases for proteins is an alternative to promote the significant identification of peptides. The development of specific DB for plant antimicrobial peptides, with information about peptide sequences, functional genomic data, structural motifs and domains of molecules, functional domains, and peptide-biomolecule interactions are valuable and necessary.

**Electronic supplementary material:**

The online version of this article (doi:10.1186/s12864-016-3332-8) contains supplementary material, which is available to authorized users.

## Background

In agriculture, alternative control of pests and diseases is strategic in attention to food security and environmental preservation [1]. The emergence of resistant microorganisms to conventional plant protection encourages the search for agents with antimicrobial action modes that are under-exploited [2], such as antimicrobial peptides (AMPs). Plant AMPs are ubiquitous in nature, present a broad spectrum and fast action against microorganisms and low cytotoxicity to animals. They have a potential antimicrobial effect that does not harm the environment [3]. Plant AMPs can act against fungi, bacteria and enveloped viruses, inhibit hydrolases from insects [4, 5] and act as anti-viral, anti-tumor, anti-inflammatory, healing and immunomodulators [6].

Most plant peptides are linear, smaller than 10 kDa, rich in cysteine, cationic and partially hydrophobic [4, 7]. The main families even now characterized are Defensins [8–10], Thionins [11], Lipid transfer proteins (LTPs) [12], Cyclotides [13], Hevein-like peptides [14], Knottin-like peptides [15], MBP-1 [16], Snakins [13], Shepherdins [17], 2S Albumin-like peptides [13], Ib-AMP1 [18], and Small proteinase inhibitors [11]. Some works show in detail the structures, functions and uses of plant AMPs [8, 15, 19–21]. Defensins, Thionins, LTPs and Hevein-like peptides are being widely investigated.

Hevein-like peptides have recently attracted interest of scientists, with a large number of reports in the last five years, such as the identification of Hevein-like domains containing 10 Cys in wheat [22]; the differential Cys-distribution in Hevein-like in *Taraxacum officinale* [23]; the study of genes encoding Hevein-like in wheat [24]; the inhibition of fungal metalloproteinase by Hevein-like from wheat [25]; *in silico* studies to identify novel precursors of Hevein-like [26]; and studies of networks of interaction involving plant AMPs, not only Hevein-like [27].

Hevein-like peptides are described as promising for biotechnological applications [13, 19, 28], including in agribusiness and for animal therapy [29]. Odintsova et al. [30] reported the action of Hevein-like peptides of *Pharbitis nill* in controlling the growth of eight pathogenic fungi in low concentrations (IC_50_ from 0.1 to 3.7 μM). Heterologous expression of Hevein-like peptides successfully increased the resistance of the transformed organisms to fungi, such as *Escherichia coli* [31], tomato plants [32], tobacco and arabidopsis plants [33]. Plants can be enriched in Hevein-like peptides for the extraction of natural pesticides [34] or to produce genotypes that are resistant to pathogens. The alternative control of pests and diseases in agriculture is strategic for food security and environmental preservation [1].

The study of AMPs is however hampered by its small size. Proteomics tools applied to peptides [35] are able to successfully isolate AMPs, despite the low concentrations of the peptides in the tissues. However, computational tools need to be adjusted to allow significant identification of the peptides by the alignment of short peptide sequences (up to 9 kDa). Low E-values for sequence alignments are desirable, since E-value depends inversely on the length of the query sequence and directly on the length of the database [36]. These tools are valuable to prospect and characterize plant AMPs for academic and/or biotechnological purposes.

Species of *Capsicum* sp. genus are widely reported as AMPs producers. In agribusiness, bell pepper (*Capsicum annuum*) plants are regarded as waste after fruits harvesting. However they produce compounds that have not yet been exploited and are biotechnologically interesting. This work reports the isolation using peptidomics tools, and the identification and partially characterization using computational tools of an antimicrobial peptide from bell pepper (‘Magali R’) leaves, and evidenced the usefulness of records and the in silico analysis for the study of plant AMPs aiming a future application in biotechnological research.

## Methods

### Materials and reagents

Materials and reagents were purchased from Sigma-Aldrich (St. Louis, Missouri, USA), GE Healthcare (GE Corporate, Wisconsin, USA), Bio-Rad (Hercules, California, USA), Merck (Kenilworth, New Jersey, USA), and other companies offering high quality products. For chromatography and mass spectrometry, materials were of spectrometric grade. Specific reagents are mentioned ahead.

### Plant material and microorganisms

Bell pepper (*Capsicum annuum* L. ‘Magali R’) plants (*n* = 120) were cultivated in June-July 2011, in a greenhouse at Universidade Federal de Viçosa (UFV), Brazil (20°45′ S, 42°52′ W, 690 m a.s.l.). Cultivation were in a greenhouse under natural light at > 85% relative humidity, from 20 °C to 38 °C with an 11 h photoperiod at 1.0 m above the floor. All fully-expanded leaves from 60-d-old plants were harvested as peptide source and maintained at −80 °C until use. The phytopathogenic test-bacteria *Clavibacter michiganensis* ssp. *michiganensis* (Gram-positive) and *Ralstonia solanacearum* (Gram-negative), provided by the Department of Plant Pathology at UFV, were maintained under 30% (v/v) glycerol, and grown according to Teixeira et al. [37].

### Protein extraction, ultrafiltration and quantification

Frozen leaves (150 g) were powdered and extracted with 600 ml of 50 mM Tris-HCl, pH 7.0, containing the protease inhibitors 10 mM ethylenediamine-tetraacetic acid, 1.0 mM phenylmethylsulphonyl fluoride, 1.0 mM benzamidine, and 2.0 mM thiourea [37]. After centrifugation at 20,300 × *g* for 30 min at 4 °C, the supernatant was fractionated using saturated ammonium sulfate at 35 and 75%, sequentially, respectively for 2 and 16 h at 4 °C, and centrifuged as above. The precipitate was resuspended in 200 ml of 50 mM Tris-HCl, pH 7.0, and the fraction was ultrafiltered using two AMICON membranes (10-kDa and 1-kDa; Millipore, Billerica, USA), producing the fraction labeled SE1-10. The soluble protein was determined by bicinchoninic acid method [38], using bovine serum albumin as standard protein. Three replicates have been made.

### Peptide isolation by chromatography and electrophoresis

Chromatography experiments were carried out using a Waters System (Waters Corporation, USA). The fraction SE1-10 (500 μL) was separated by reverse-phase chromatography (RPC) in C18-column Shim-pack CLC-ODS (4.6 × 150 mm, 5.0 μm, Shimadzu, Japan) or in C4-column YMC-Pack (4.6 × 250 mm, 5.0 μm, YMC Co., Kioto, Japan). The columns were equilibrated with 0.1% (v/v) TFA (Solution A) and peptides were eluted in increasing gradient of 0.1% (v/v) TFA and 80% (v/v) acetonitrile (Solution B), at 1.0 mL.min^−1^. In RPC-C18, peaks were collected at 1.0 mL.min^−1^; in RPC-C4, 200-μL fractions were collected along 90 min at 0.5 mL.min^−1^. The molecular exclusion chromatography (MEC) was performed in the column Protein Pack 60 Å (7.8 × 300 mm, 5.0 μm, separation range 2–10 kDa, Waters Corporation, USA). The column was equilibrated and eluted in 25 mM Tris-HCl, pH 7.0, added of 250 mM NaCl, at 0.1 mL.min^−1^. For all separations, chromatographic profiles were followed by absorbance at 214 nm.

SDS-Tricine-PAGE [39] was used to check the degree of peptide enrichment after ultrafiltration and RPC. Samples were boiled for 10-min in loading buffer and separated in a three-phase gel. Broad Range Molecular Weight Standards (161-0317, Bio-Rad, Hercules, USA) and Ultra-Low Range Molecular Weight Standards (M3546, SIGMA, USA) were used. Peptide bands were stained with Coomassie Brilliant Blue G-250 [40].

### Peptide reduction, alkylation and proteolysis

Prior to mass spectrometry analyzes the peptide samples were transferred to siliconized PCR tubes (200 μL) previously washed with methanol, and reduced with 50 mM dithiothreitol in 100 mM ammonium bicarbonate, pH 8.0, for 1 h at 70 °C, in a thermomixer at 500 rpm. After this step, the peptides were alkylated with 100 mM iodoacetamide in 100 mM ammonium bicarbonate, pH 8.0, for 1 h at 28 °C, in the absence of light, in a thermomixer at 500 rpm. To determine the number of cysteine radical free or involved in disulfide bonds, the peptide sample was analyzed by mass spectrometry in three conditions: native sample, reduced/alkylated sample, and not-reduced/alkylated sample. Peptide mass-values were compared for the samples, considering that alkylation with acetamide added 57.05 Da for each alkylated sulfhydryl group [41].

For the tryptic digestion [42], Trypsin Gold V5280, mass spectrometry grade (Promega, Madison, Wisconsin, USA) was used at 0.025 μg.μL^−1^ solubilized in 40 mM ammonium bicarbonate, pH 8.0, and 10% (v/v) acetonitrile. The proteolysis carried out at 37 °C for 16 h. Alternatively, aiming to follow peptide degradation, proteolysis was performed by a larger period, with collecting samples at 6, 12, 24, and 36 h. Before mass spectrometry (MS) analysis, samples were concentrated in a speedvac and desalted using ZipTip C18-columns (Millipore, Billerica, MA, USA).

### Mass spectrometry and peptide identification

Mass spectra of the native peptide or the tryptic peptides were obtained on a matrix-assisted laser desorption/ionization (MALDI) tandem time-of-flight (TOF/TOF) mass spectrometer (MALDI-TOF/TOF) model Ultraflex III (Bruker Daltonics, Bremen, Germany), in the positive ion reflector mode. The samples were mixed with 5 μg.mL^−1^ α-cyano-4-hydroxycinnamic acid (Bruker Daltonics, Bremen, Germany) in a proportion of 1:3 (sample:matrix) and dispensed onto an MTP AnchorChip MTP 600/384 TF target (Bruker Daltonics, Bremen, Germany). Calibration was done externally with the Peptide Standard Calibration II Mixture (Bruker Daltonics, Bremen, Germany). For MS1, the methods of analysis were LPPepMix (500 to 5000 Da) and LPProtMix (3000 to 20,000 Da), and for MS2, *LIFT* technology (40 to 1878 Da) was performed. The spectra were processed using flexAnalysis software Version 3.0 (Bruker Daltonics, Bremen, Germany, PN246503) and peak lists (xml and mgf format) were used for identification of the proteins using the peptide mass fingerprinting (PMF) method and by peptide fragment fingerprinting (PFF), both using the Mascot software [43] for Green Plants or All entries and NCBInr [44] and Swiss-Prot [45] protein Databases. For the search, the mass tolerances for the parental ions and fragment ions were set to 50 ppm and 0.1 Da, respectively. Peptides were searched using fully tryptic cleavage constraints and up to one missed cleavage sites was allowed, fixed modification for carbamidomethylation of Cys residues and variable modification for oxidation of methionine residues. For significant identification, the criteria used for PMF were a significant Mascot score (*p*-value < 0.05) for at least four peptides showing matches and greater than 20% sequence coverage; and for PFF, significant Mascot alignment score (*p*-value < 0.05) for at least two peptides. Amino acid sequencing in MS2 spectra were confirmed by manual de novo sequencing [46].

The N-terminal amino acid residues of the peptide were identified by automated Edman degradation sequencing in a peptide sequencer (PPSQ-33 Shimadzu, Japan). The equipment was calibrated with phenylthiohydantoin-amino acids at 25 pmol, samples were reduced, alkylated, desalted by RPC-C18, concentrated, and analyzed onto a glass fiber membrane previously treated with polybrene. Samples were alternatively analyzed onto a polyvinylidene fluoride membrane. Analyses were processed with PPSQ-30 Data Processing Software.

### Determination of peptide sequence

Computational tools and databases (DB) of protein and peptide sequences were used alternately to compose the initial sequence of the peptide using mass spectrometry data and sequentially to unravel the complete sequence of the peptide. Portals were the ExPASy server and the Bioactive Peptide Databases [47], and The Pepper Genome Database (release 2.0) [48]. Databases for proteins were NCBI-Protein DB [49], NCBI-GenBank DB [50], NCBI-Reference Sequence (RefSeq) DB (nr) [44], UniProtKB/Swiss-Prot DB [51], and Swiss-Prot Protein DB [45]. Databases for peptides were Antimicrobial Peptide Search - APD2 [52], Database Linking AMPs [53], AMPer [54], CAMP [55], and PhytAMP DB [56]. Softwares used for peptide characterization were ClustalOmega [57] to produce multiple sequence alignment; Pairwise Sequence Alignment [58] to align two sequences; NCBI-Protein BLAST [59] to compare primary amino acid sequence information to identify the query sequence; NCBI-PSI-BLAST [60] to refine comparison of protein sequence alignment to find evolutionary relationships; PeptideCutter [61] to predict potential cleavage sites of trypsin action and then produce a theoretical trypsinolysis, and ProtParam [61] to compute physical and chemical parameters of the peptide molecule.

### Antimicrobial assays

The inhibitory activity of the peptide-enriched fraction was evaluated against the plant pathogens *Ralstonia solanacearum* (Gram-negative) and *Clavibacter michiganensis* ssp. *michiganensis* (Gram-positive) by using the Microplate Test at 560 nm [37]. Tests were carried out in liquid LB culture medium at 28 °C, in three biological replicates.

## Results

### Isolation of a plant peptide using proteomics procedures

**Fig. 1:**
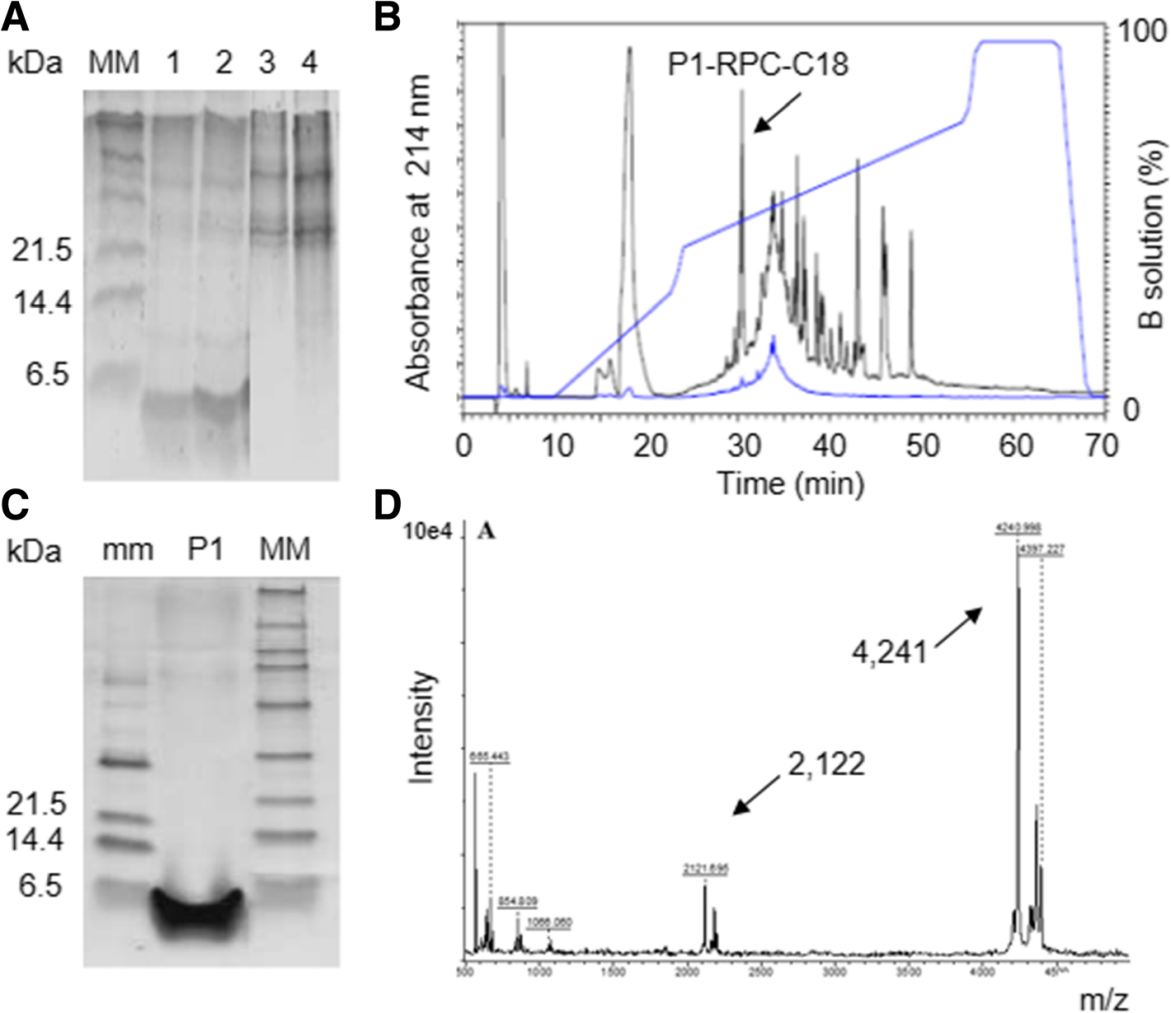
Isolation of a peptide enriched fraction from bell pepper leaves using reverse-phase chromatography (RPC). **a** SDS-Tricine-PAGE of the peptide fraction SE1-10 (lanes 1, 2) and protein fraction (lanes 3, 4); **b** RPC-C18 profile of the fraction SE1-10, producing P1-RPC-C18 (*arrow*); **c** SDS-Tricine-PAGE of the fraction P1-RPC-C18j; **d** Mass spectrometry profile of the fraction P1-RPC-C18, showing the mono-charged (4241 Da) and the doubly charged (2122 Da) peptide ions. MM: Broad range molecular marker; mm: Low range molecular marker

Fractionation of the soluble extract (SE) of leaves from bell pepper with ammonium sulfate (between 35 and 75% saturation), followed by ultrafiltration procedure (in membranes with molecular weight cut-off of 1 and 10 kDa) produced the fraction SE1-10 enriched in constitutive peptides ranging from 4 to 5 kDa. SDS-Tricine-PAGE showed peptides in the fraction SE1-10 and the absence of these peptides in the fraction containing proteins larger than 10 kDa (Fig. 1a). Reverse-phase chromatography (RPC) in C18-column (RPC-C18) evidenced the complexity of SE1-10 and isolated the peptide fraction P1-RPC-C18 (Fig. 1b and c). Mass spectrometry (MS) analysis has detected an interest mono-charged peptide with 4241 Da, and doubly charged with 2122 Da (Fig. 1d).

**Fig. 2:**
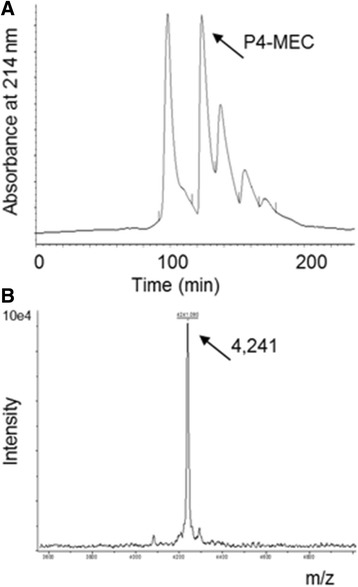
Isolation of a peptide enriched fraction from bell pepper leaves using molecular exclusion chromatography (MEC). **a** MEC profile using a Protein Pack 60 Å column (7.8 × 300 mm, 5.0 μm, 2–10 kDa) of the fraction P1-RPC-C18; **b** Mass spectrometry profile of the fraction P4-MEC (*arrow*) showing the mono-charged (4241 Da) and the doubly charged (2122 Da) peptide-ions. The chromatography was performed in isocratic conditions using 25 mM Tris-HCl, pH 7.0, and 250 mM NaCl at 0.1 ml.min^−1^. Peaks were collected at 1.0 mL.min^−1^

Targeting a higher purity of these peptides, P1-RPC-C18 was fractionated by three chromatographic procedures. RPC in C4-column (RPC-C4) isolated two fractions (P2-RPC-C4A and P2-RPC-C4B), which contained the ions 4240 and 2122 Da (see Additional file 1). A RP rechromatography on C18-column produced a fraction containing the same two ions (see Additional file 2), similarly to the Fig. 1d. The molecular exclusion chromatography (MEC) produced fractions with the highest degree of purity (Fig. 2a). The ion 4240 Da was detected in fraction P4-MEC, the most purified (Fig. 2b).

Fraction P4-MEC was then exploited to identify and characterize the peptide of 4240 Da. The alkylation with dithiothreitol of two aliquots of the fraction P4-MEC, with previous reduction of only one aliquot, demonstrated the presence of eight cysteine residues involved in four disulfide bonds. MS analysis has detected the mass of 4706 Da for the reduced peptide (see Additional file 3), considering addition of 57.05 Da of iodoacetamide to eight reduced and alkylated sulfhydryl radical. This peptide could be member of one of three families of plant natural peptides, which are Defensins, Thionins, or Heveins, presenting about 4–5 kDa and four disulfide bonds [4].

Automatic Edman sequencing of the P4-MEC fraction, by using liquid sample or membrane, indicated the N-terminal peptide sequence as 1QN_ _RQAGGR10, although in low yield. The scarcity and the difficulty of obtaining peptide sample with high purity led to the search of computer tools in association with MS analysis aiming to characterize the isolated peptide.

### Peptide identification by mass spectrometry and computational tools

**Fig. 3:**
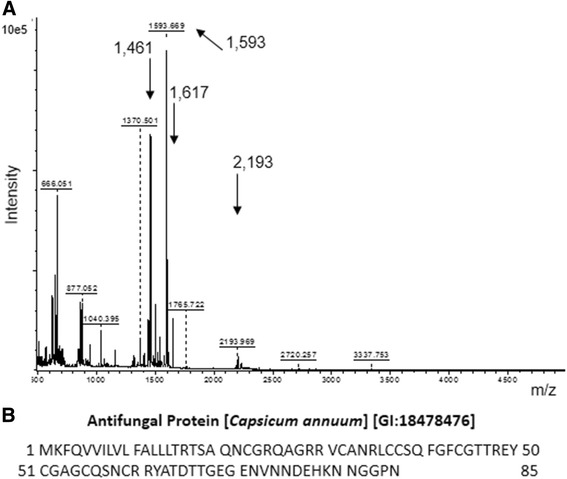
Mass spectrometry (MS1) analysis of the peptide fraction P4-MEC after trypsinization. **a** MS1 profile obtained in MALDI-TOF/TOF; **b** Sequence of the Antifungal Protein [*Capsicum annuum*] [UniProtKB:Q8W2B2_CAPAN] identified by peptide mass fingerprinting, although as not-significant (for *P* < 0.05). Ions with m/z-values of 1461; 1593; 1617; and 2193 Da matched fragments of the Antifungal Protein at positions 31 to 60 after the theoretical trypsinolysis using PeptideCutter software

Aliquots of the P4-MEC fraction after trypsinolysis and analysis on MALDI-TOF/TOF by MS1 spectrum (Fig. 3a), using the PMF procedure [62] and FlexAnalysis (Bruker) and Mascot tools, resulted in the identification of the Antifungal Protein [*C. annuum*] [GI:18478476, GenBank:AAL73184.1, UniProtKB:Q8W2B2_CAPAN], with 9748 Da and pI 8.73, but not significant (for *P* < 0.05). The region with sequence similarity corresponds to residues from 31 to 60 of the Antifungal Protein, which has 85 amino acid residues (Fig. 3b).

In silico proteolysis of the Antifungal Protein [GI:18478476] sequence using the PeptideCuttter software has produced fragments with mass values identical to the ion masses obtained in MS1 spectrum of the peptide in question: 1461.6; 1593.8; 1617.6; and 2194.5 Da (Table 1). ProtParam software confirmed the mass-values of the theoretical peptide sequences, alkylated or not, by confrontation with mass values of the MS1 spectrum. The MS2 spectra of the four ions were obtained and automatically analyzed by Peptide Fragment Fingerprint (PFF) [62] and MASCOT tools, and showed no significant identification of proteins. Ions in the MS2 spectra were also analyzed by manual de novo sequencing, and the sequences of these ions have matched with the sequences of fragments of the same mass, which were obtained by *in silico* proteolysis, in positions between 31 and 60 of the Antifungal Protein [GI:18478476].

**Table 1:** Ionic fragments obtained from the theoretical cleavage of the Antifungal Protein using PeptideCutter

Sequence^a^	MW (Da) (not alkylated)	Cys^b^ number (x 57.05 Da)^c^	MW (Da) (alkylated)^c^
31 VCANR 35	561.7	1	618.1
36 LCCSQFGFCGTTR 48	1422.7	3	1593.8
31 VCANRLCCSQFGFCGTTR 48	1966.3	4	2194.5
49 EYCGAGCQSNCR 60	1290.4	3	1461.6
49 EYCGAGCQSNCRR 61	1446.5	3	1617.6

^a^According to Antifungal Protein [GI:18478476] sequence

^b^Cys, cysteine residue

^c^The mass-value of 57.05 Da is added in the alkylation event of each sulfhydryl group of the cysteine residue

P4-MEC fraction was again trypsinized targeting a significant identification of the peptide. Samples were collected at 6, 12, 24, and 36 h after proteolysis, and MS1 and MS2 spectra were analyzed for all samples. Using the PMF tool, the Antifungal Protein [*C. annuum*] [GI:18478476] and Chitin Biding Protein [*C. annuum*] [GI:169930135, GenBank:ACB05666.1] with 9718 Da and pI 8.93 were identified in the sample with 12 h of trypsinolysis in the NCBI database, but not with a significant sequence similarity although with 35% coverage and 3 matches. In analysis of the MS2 spectra by PFF, the identification of the antifungal protein was significant in NCBI DB for two fragments in all trypsinization times. For the fragment of 1461 Da, the analysis time and score/threshold-values (*P* <0.05) were: 6 h, 82/31; 12 h, 87/42; 24 h, 92/39; and 36 h; 52/38. Similarly for the fragment of 1593 Da: 6 h, 87/34; 12 h, 88/33; 24 h, 86/33; and 36 h, 87/34. Manual de novo sequencing for these two ions and for ions with 1290 and 1502 Da in MS1 spectrum confirmed the previously found sequences (Table 2).

**Table 2:** De novo sequencing of ions of the P4-MEC fraction after controlled trypsinolysis^a^

Ion (m/z)^b^	Sequence	Modifications^c^
1593	36 LCCSQFGFCGTTR 48	Alkylated Cys
1502	31 VCANRLCCSQFGFCGTTR 48	Alkylated Cys and R48 partially degraded
1290	49 EYCGAGCQSNCR 60	Not-alkylated Cys
1461	49 EYCGAGCQSNCR 60	Alkylated Cys

^a^Trypsinolysis was developed by 6, 12, 24, and 36 h in P4-MEC fraction and the ions were analyzed by mass spectrometry

^b^All ions were detected in all MS2 spectra for all trypsinolysis times

^c^The mass-value of 57.05 Da is added in the alkylation event for each sulfhydryl group of the cysteine residue

**Fig. 4:**
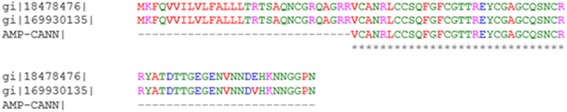
Alignment of the proposed sequence for AMP-CANN after tandem mass spectrometry and de novo sequencing. Identity for 30 amino acid residues was detected with the Antifungal Protein [*Capsicum annuum*] [GI:18478476] and the Chitin Biding Protein [*Capsicum annuum*] [GI:169930135] at positions 31 to 60

**Fig. 5:**
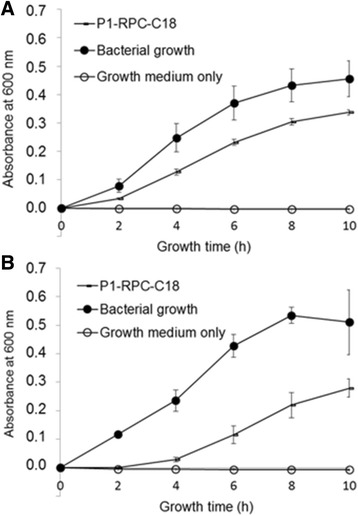
Antimicrobial activity of the peptide fraction P1-RFC-C18. The antimicrobial activity assays of the peptide fraction were against the phytopathogenic bacteria *Clavibacter michiganensis* ssp. *michiganensis* (Gram-positive, **a**) and *Rasltonia solanacearum* (Gram-negative, **b**)

Then, we propose the partial sequence of the peptide, with 30 residues, named as AMP-CANN (Fig. 4). AMP-CANN refers to “antimicrobial peptide from *C. annuum*” since Games et al. [63], in our laboratory have shown that these fractions, enriched in peptide by fractionation with ammonium sulfate and chromatography, partially controlled the in vitro growth of the phytopathogenic bacteria *Ralstonia solanacearum*, *Clavibacter michiganensis* ssp. *michiganensis* and *Erwinia carotovora* ssp*. carotovora*, and of the fungus *Alternaria solani.* Growth inhibition assays were also successfully performed with P1-RPC-C18 fraction for *Ralstonia solanacearum* (Gram-negative) and *Clavibacter michiganensis* ssp. *michiganensis* (Gram-positive) (Fig. 5).

The sequence of 30 residues was used to identify the peptide in databases (DB) (Table 3). Analysis using Blast tool in non-redundant (nr) NCBI-RefSeq DB showed sequence similarity (*P* < 0.05) for six proteins containing chitin binding domain (CBD). Similarly, in UniProtKB, eleven sequences of heveins and CBD of *Hevea brasiliensis* (HEVBR) were identified, seven of which showed aligned regions with the sequence of AMP-CANN. Thereby, this peptide is a hevein-like extracted from *C. annuum*, and will be called HEV-CANN from now on.

**Table 3:** Bioinformatics tolls and sites mentioned in the text

Name	Link	Reference
Softwares		
ClustalOmega	http://www.ebi.ac.uk/Tools/msa/clustalo/	[57]
NCBI/Protein BLAST	http://blast.ncbi.nlm.nih.gov/Blast.cgi	[59]
NCBI/PSI-BLAST	http://blast.ncbi.nlm.nih.gov/Blast.cgi	[60]
Pairwise Sequence Alignment	http://www.ebi.ac.uk/Tools/psa/	[58]
PeptideCutter	http://web.expasy.org/peptide_cutter/	[61]
ProtParam	http://web.expasy.org/protparam/	[61]
Peptide databases		
AMPer	https://omictools.com/amper-tool	[54]
Antimicrobial Peptide Search (APD2)	http://aps.unmc.edu/AP/	[52]
CAMP	http://www.bicnirrh.res.in/database.php	[55]
Database Linking AMPs (LAMP)	http://lampdatabase.com/	[53]
PhytAMP DB	http://phytamp.pfba-lab-tun.org/main.php	[56]
Protein database		
Mascot	http://www.matrixscience.com	[43]
NCBI-Conserved Domain DB (CDD)	http://www.ncbi.nlm.nih.gov/Structure/cdd/cdd.shtml	[64]
NCBI-Protein DB	http://www.ncbi.nlm.nih.gov/protein	[49]
NCBI-GenBank DB	http://www.ncbi.nlm.nih.gov/genbank/	[50]
Swiss-Prot Protein DB	http://web.expasy.org/docs/swiss-prot_guideline.html	[45]
UniProtKB/Swiss-Prot	http://www.uniprot.org/	[51]
NCBI-Reference Sequence (RefSeq) DB (nr)	http://www.ncbi.nlm.nih.gov/RefSeq/	[44]
Portal		
ExPASy	http://www.expasy.org/	[61]
Bioactive Peptide DBs	http://www.uwm.edu.pl/biochemia/index.php/pl/biopep/32-bioactive-peptide-databases	[47]
The Pepper Genome DB (release 2.0)	http://peppersequence.genomics.cn/page/species/index.jsp	[48]

In order to complete the sequencing of the peptide, searches were conducted aligning sequences in the specific DB Antimicrobial Peptide Search (APD2) and Database Linking MPAs, without successful results about similarity in general or for species-specific searches. In the portal Bioactive Peptide Databases, Amper DB detected alignment (*P* < 0.05) with two mature Hevein fragments from *Hevea brasiliensis* and *Eucommia ulmoides*; CAMP found 16 Hevein-like and/or CBD-protein that showed significant alignments (E-value from 2e-16 to 6e-8). PhytAMP DB showed significant alignment for HEV-CANN with the sequence of the antimicrobial peptide Hevein from *Hevea brasiliensis* [PhytAMP:PHYT00231, HEV-PHYT00231], which is 43 amino acid residues and corresponds to the residues between 18 and 60 of the Pro-Hevein protein [UniProt:P02877-HEVE_HEVBR], with 204 residues.

**Fig. 6:**
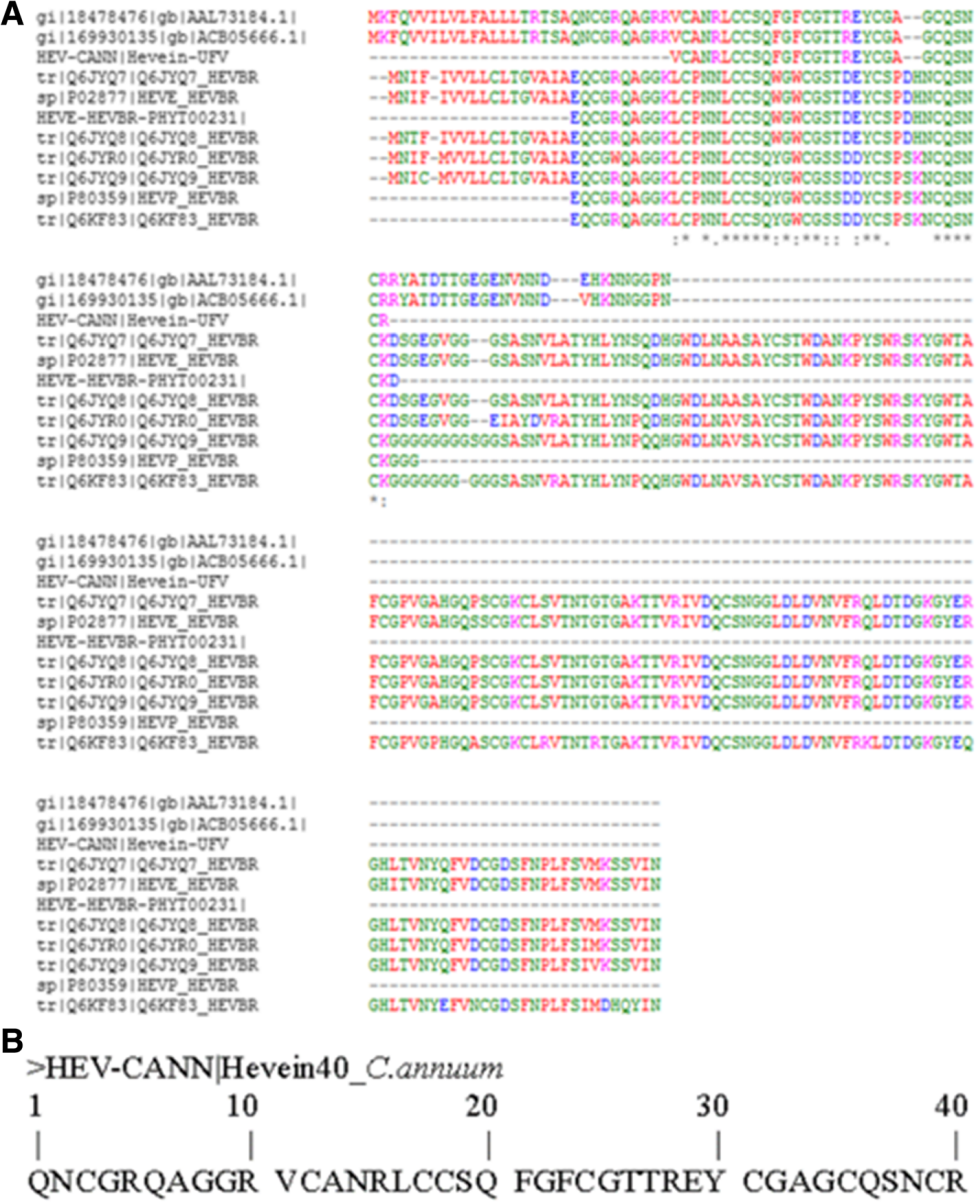
HEV-CANN and other Hevein-like sequences. **a** The proteins with sequences aligned with HEV-CANN were the Antifungal Protein [GI:18478476] and the Chitin Biding Protein [GI:169930135], both from *Capsicum annuum*; the peptide Hevein (HEV-PHYT00231); and seven Hevein-like from *Hevea brasiliensis*. **b** The complete sequence of HEV-CANN

Alignment of HEV-CANN, HEV-PHYT00231, seven sequences of HEVBR and two sequences of *C. annuum*, which had presented significant match with HEV-CANN (Fig. 6a), showed a high similarity in the region corresponding to the CBD, near the amino-terminal region of the major sequences. Comparing the residues obtained in the automatic sequencing (1QN_ _RQAGGR10) with the correspondent residues in Fig. 6a, we suggest that the initial HEV-CANN sequence is 1QNCGRQAGGR10 (Fig. 6b).

Analysis of the complete HEV-CANN sequence using ProtParam software indicated that it was a stable peptide with 40 amino acid residues, eight-Cys residues, and 4258.8 Da since 8 Da were deducted by the presence of four disulfide bonds. Theoretical pI-value was 8.78 and grand average of hydropathicity (GRAVY) was −0.465, with five positively charged residues and one negatively charged residue, corresponding to a cationic peptide. HEV-CANN is a stable peptide, with instability index (II) computed to be 23.99/40. HEV-CANN characteristics are partially according to those of Hevein peptide from *Hevea brasiliensis*, HEV-PHYT00231 [PhyTAMP:PHYT00231, UniProt:P02877, EMBL:M36986], with 43 amino acid residues, 4727.1 Da, eight cysteine residues, four disulfide bonds, theoretical pI-value of 4.83, and GRAVY-value of −1.019, three positively charged residues and five negatively charged residues. HEV-PHYT00231 was classified as unstable, with instability index (II) computed to be 46.41/40.

HEV-CANN and HEV-PHYT00231 showed alignment with the conserved region of the CBD of the Pro-Hevein with 204 residues [UniProt:P02877-HEVE_HEVBR], near the amino-terminus of members of ChtBD1-Superfamily [CDD:cl16916], with E-value of 4.89e-08 for the alignment (see Additional file 4). HEV-CANN proved to be a member of the Superfamily Hevein [64], also called type 1 chitin-binding-domain [CDD:cl16916].

The search for alignment of HEV-CANN sequence using the PSI-BLAST tool, which provides a means of detecting distant relationships between proteins from different sources, showed 963 results for alignment with e-values lower than the defined threshold 0f 0.005. The best alignment for all tested rounds were for chitin-binding protein [*C. annuum*] [GenBank:ACB05666.1] and antifungal protein [*C. annuum*] [GenBank:AAL73184.1], both presenting identical parameters that are total/maximum score 79/79, 98% identity, 100% query cover, and E-value of 2e-17. For *C. annuum*, the five hits obtained showed proteins related to plant defense, which are chitin binding protein [GenBank:ACB05666.1], E-value of 2e-17; antifungal protein [GenBank:AAL73184.1], E-value of 2e-17; wound-induced protein CBP1 precursor [GenBank:AAF18934.1], E-value: 1e-06, pathogenesis-related protein 4b [GenBank:AEI52548.1], E-value: 2e-06, and chitinase [GenBank:ACM47315.1], E-value: 4e-06. Most proteins have been identified as class I chitinases; some were class 4 pathogenesis-related protein (PR-4) (see Additional file 4), other antifungal proteins, wound-induced protein, and/or Hevein-like proteins. For *Solanum tuberosum* (potato) and *Solanum lycopersicum* (tomato), 22 hits (E-values: 5e-10 to 8e-6) and 7 hits (E-values: 5e-8 to 9e-6), respectively, showed alignment with chitinases or wound-induced proteins. All detected protein classes are involved in plant defense against phytopathogens.

Searches for sequence alignments were also carried out at The Pepper Genome Database (release 2.0), using the database Capsicum.annuum.var.glabriusculum_Chiltepin_v2.0_PEP.fasta, containing 34,476 sequences, and recovered 10 sequences producing significant alignments and E-values as low as from 5e^−^13 to 8e^−^8. The identified proteins were chitinases, chitin-binding proteins, antifungal proteins, heveins, and/or PR-4 proteins, similarly to those found for general databases, indicating that HEV-CANN are involved in plant defense against phytopathogens.

## Discussion

HEV-CANN, an antimicrobial Hevein-like peptide with 4.26 kDa and pI-value of 8.8 was isolated in this work from bell pepper leaves and characterized as a member of the type-1 chitin-binding domain superfamily (ChtBD1-Superfamily) in plants [26]. Hevein-like peptides have a CBD, also called Hevein-domain, which is characteristic of the plant kingdom and exhibit antifungal activities. The binding of CBD to chitin reduces or stops the elongation of the cell wall of fungi. CDB is present in multi-domain PR-proteins [65] such as chitinase (classes I and IV of the PR-3 family, and Class I of the PR-4 family), agglutinins and lectins. Archer [66] reported the first antimicrobial peptide from Hevein class, HEV-PHYT00231, isolated from *Hevea brasiliensis*, with 4.73 kDa and pI-value of 4.8. The peptide HEV-PHYT00231 corresponds to the CBD region of Pro-Hevein [UniProt:P02877] and other chitin-binding proteins. Since chitin is essential for the skeleton of the fungal cell wall, and is not present in vertebrates, CBD offers specificity to Hevein-like proteins or peptides in controlling fungal pathogenesis.

HEV-CANN also presented activity against Gram-positive and Gram-negative bacteria, while HEV-PHYT00231 was not able to inhibited Gram-negative bacteria [67]. Some Hevein-like peptide control the growth of bacteria or fungi chitin-free with participation of the cationic and amphipathic surface [26, 68]. HEV-CANN is a cationic molecule and present five positive charges on surface and HEV-PHYT00231 is anionic and has three positive charges on the surface; both are classified as plant antimicrobial peptides (AMPs).

Although evolutionarily ancient, the wide range of plant AMPs is poorly understood regarding the structural, biological and functional properties, distribution profile in the source organisms and tissue expression profile [23]. HEV-CANN has shown to be a member of the constitutive defense in bell pepper leaves, which is valuable for the study and application if compared with induced AMPs. Constitutive AMPs are not expressed transiently, reducing difficulties of recovering the peptides during the separation from contaminants. Proteomic and computational tools have been successfully employed in this work to isolate, identify and/or partially characterize HEV-CANN, which has shown promising features to biotechnology exploitation.

HEV-CANN showed up as a soluble peptide under hydrophilic conditions, according to GRAVY-value. The aggregation that is characteristic of many vegetable AMPs generates losses during isolation. Aggregates presenting high molecular weight are removed from the peptide samples together with the larger proteins, which was not observed in this study (Fig. 1a). The high solubility of HEV-CANN allowed the efficient use of ultrafiltration equipment in this work. Industrial versions of ultrafiltration devices are available and can be successfully used to enrich the peptide in a stepwise process. Cross-flow filtration equipment are also available and perform this task efficiently [69].

HEV-CANN presented other characteristics that favor the study. Ease of HEV-CANN ionization in MS analysis yielded spectra with high intensities and defined peaks, which ensured the success of the fragmentation analyses for MS2 and the completion of the manual de novo sequencing for the ions used to unravel the primary sequence of the peptide. The high stability of HEV-CANN, with index less than 40 [70], also favors the study of this peptide and is possibly influenced by the presence of four disulfide bonds in the small molecule.

Careful sample preparation, isolation with high efficiency equipment, mass spectrometry and computational tools are generally indispensable to the study and exploration of unknown bioactive peptides. This work highlighted the usefulness of records and in silico analysis for the study of plant AMPs. However, the computational tools for proteomics and databases are not adjusted for short sequences, which hinders the significant identification of plant peptides smaller than 10 kDa. E-value for sequence alignments depends directly on the length of the database and inversely the length of the query sequence [36]. Consequently, it became evident that exists a necessity to adjust or develop computational tools, and also build specific databases to assist in the identification and characterization of plant AMPs for academic and/or biotechnological purposes.

The Pepper Genome Database (release 2.0), specific to *C. annuum* sequence data, rendered significant alignments for proteins but not for peptides. CAMP, a DB for antimicrobial peptides, identified sequences corresponding to larger proteins, and only PhytAMP DB identified significant alignment for a peptide sequence. Therefore, it is essential to adjust or develop computational tools to allow significant identification of peptides despite their small amino acid sequences. Statistical analysis should consider that as short sequences as the HEV-CANN can match the full sequence of a peptide. Information of secondary structure, tertiary and functional domains are also desirable in peptide databases, since not always the primary sequence is highly conserved.

The success of heterologous expression of Hevein-like peptides in heterologous bacterial or plant models [31–33] allows us to view HEV-CANN as promising in the transformation to enhance the constitutive protection of plants and thus develop genotypes more resistant to disease. An alternative is to express the peptide in the same plant species or closely related species and generate cisgenic plants [71]. Natural agrochemicals [34] can be produced from plant extracts enriched in HEV-CANN. Possible uses are for plants grown in greenhouses or in post-harvest agricultural products (fruits, vegetables, and grains) to increase shelf life, or marketable seed protection. Smart packaging or waxes containing defense peptides such as HEV-CANN, could control pathogens, reduce transpiration and give better appearance to agricultural products, improving marketing [72]. Nanotechnology, expanding, will offer methods of encapsulation to better distribute AMPS in plant tissues, with slow and steady release [73]. The plant molecular farming, known as farming or biofarming, involves the heterologous expression of AMPs in plant-based systems [21] and ensures the production of the peptide on a large scale for biotechnological use.

Production of AMP in plants certainly shows great promise for biotechnological use in sectors such as agribusiness, pharmaceutical, cosmetics and food [74]. However, there are technical limitations to be solved beforehand about the AMPs, as characterization and stability of bioactive molecules, synthesis of large numbers of disulfide bonds, functionality, and production. Information about functional structures, action mechanisms, spectrum of antimicrobial activity, and structural and kinetic stability show to be essential to the development of defense agents for biofarming of AMPs. For this, peptidomics tools [35] and computational tools applied to peptidomics need to advance greatly in allowing identify and characterize as significant the native peptide sequences by aligning sequences in specific databases.

## Conclusions

The HEV-CANN peptide, purified and partially characterized in this work, is promising as antimicrobial for developing biotechnological defense agents. HEV-CANN is a constitutive 40-residues peptide with four disulfide bonds, cationic, soluble and stable under hydrophilic conditions. It is member of the plant AMPs Hevein-like family with a chitin-binding domains; active against Gram-positive and Gram-negative bacteria, and against a fungus-test.

Genetic modifications of HEV-CANN, including by cisgenesis, may be performed to produce plants more resistant or to develop biofarming. In biofarming, HEV-CANN can be synthesized constitutively in large scale using plant systems to express natural defense compounds for agricultural and other uses. The biotechnological application of HEV-CANN proves to be promising but the structural and functional characterization of the peptide must first be completed, which needs of large amounts of purified and active peptide. Expression in heterologous microbial system or in the same plant species is shown as an alternative of producing HEV-CANN for studies. The extraction procedures are hard and expensive because natural peptides are synthesized in very low concentrations in unmodified plants.

Peptidomics tools are scarce and proteomic tools fail to identify significant peptides, even using databases for organisms with sequenced genome. The databases are deficient in information for peptides and proteins up to 30 kDa. Thus, there is a pressing need to develop computational tools and to build specific databases to work with peptides. The AMPs are members of the innate defense of all living systems. The understanding of the defense mechanisms of organisms by AMPs action requires efficient tools. In addition, AMPs are a source of antimicrobial defense mechanisms underexplored. The bioinformatics tools are especially valuable for protein and peptide studies due to structural and/or functional similarities of these molecules among the organisms. The adjustment of statistical tests in large databases for proteins is an alternative to promote the significant identification of peptides, which are small molecules. In addition, the development of specific databases for plant AMPs, with information about amino acid sequences, transcriptomic and genomic data, structural motifs and domains, functional domains, and peptide-biomolecule interactions are valuable and necessary.

## Additional files

Additional file 1: Isolation of the peptide fraction P1-RPC-C18 by reverse-phase chromatography in a C4-column (RPC-C4) and mass spectrometry profile showing the peptide ions. (PDF 66 kb)
Additional file 2: Isolation of the peptide fraction P1-RPC18 by reverse-phase rechromatography in a C18-column (RPreC-C18) and mass spectrometry profile showing the peptide ions. (PDF 50 kb)
Additional file 3: Determination of the number of disulfide bonds in HEV-CANN by mass spectrometry analysis of the fraction P4-MEC after reduction and alkylation, and after alkylation only. (PDF 49 kb)
Additional file 4: Alignment of the HEV-CANN sequence with PR-4 proteins from plants showing the chitin-binding domain region of the peptide Hevein from *Hevea brasiliensis* (residues 18 to 60) and five sequences of pathogenesis-related proteins Class-4 (PR-4). (PDF 130 kb)

## Abbreviations

AMP: Antimicrobial peptides
CANN: *Capsicum annuum*
CBD: Chitin binding domain
GRAVY: Grand average of hydropathicity
HEVBR: *Hevea brasiliensis*
HEV-CANN: Hevein-like peptide from *Capsicum annuum*
IC_50_: Concentration for 50% growth inhibition
MALDI-TOF/TOF: Matrix-assisted laser desorption/ionization tandem time-of-flight mass spectrometer
MEC: Molecular exclusion chromatography
MS: Mass spectrometry
MS1: MS in the first analyzers
MS2: MS in tandem
P1-RPC-C18: Fraction P1 eluted from a RPC-C18
P2-RPC-C4: Fraction P2 eluted from a RPC-C4
P4-MEC: Fraction P4 eluted from a MEC
PFF: Peptide fragment fingerprinting
PMF: Peptide mass fingerprinting
RPC: Reverse-phase chromarography
RPC-C18: RPC in C18-column
RPC-C4: RPC in C4-column
SE: Soluble extract
SE1-10: SE enriched in peptides from 1–10 kDa
