# Supplementary material for: Computer aided identification of a Hevein-like antimicrobial peptide of bell pepper leaves for biotechnological use
Source: BMC Genomics. 2016 Dec 15;17(Suppl 12):999. doi: 10.1186/s12864-016-3332-8 (PMC5249031; doi:10.1186/s12864-016-3332-8)
Supplement: Additional file 1: — Isolation of the peptide fraction P1-RPC-C18 by reverse-phase chromatography in a C4-column (RPC-C4) and mass spectrometry profile showing the peptide ions. (PDF 66 kb) [file 12864_2016_3332_MOESM1_ESM.pdf]

Additional file 1

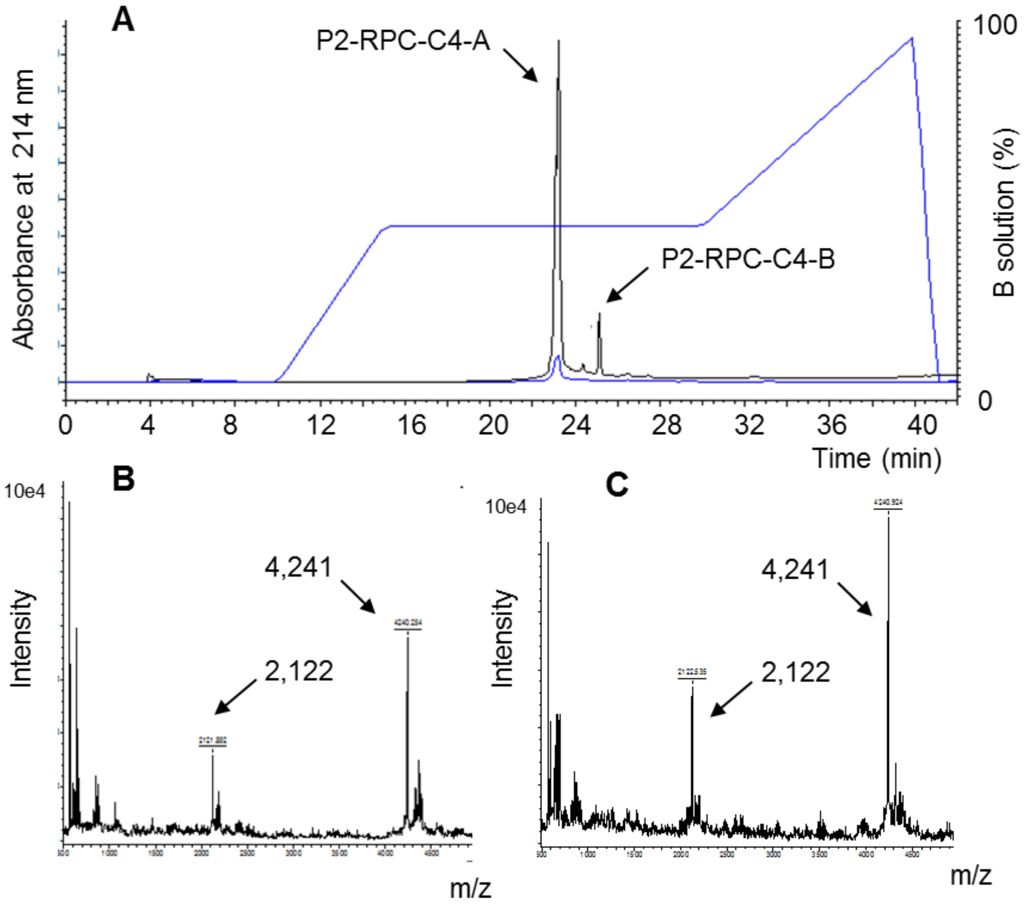

Isolation of the peptide fraction P1-RPC-C18 by reverse-phase chromatography in a C4-column (RPC-C4). (A) RPC-C4 profile of the peptide fraction P1-RPC-C18. **(B)** Mass spectrometry (MS1) profile of the fraction P2-RPC-C4A (arrow in A); **(C)** MS1 profile of the fraction P2-RPC-C4B (arrow in A), showing the mono-charged (4,241 Da) and the doubly charged (2,122 Da) peptide-ions in both profiles.
