# Supplementary material for: Computer aided identification of a Hevein-like antimicrobial peptide of bell pepper leaves for biotechnological use
Source: BMC Genomics. 2016 Dec 15;17(Suppl 12):999. doi: 10.1186/s12864-016-3332-8 (PMC5249031; doi:10.1186/s12864-016-3332-8)
Supplement: Additional file 2: — Isolation of the peptide fraction P1-RPC18 by reverse-phase rechromatography in a C18-column (RPreC-C18) and mass spectrometry profile showing the peptide ions. (PDF 50 kb) [file 12864_2016_3332_MOESM2_ESM.pdf]

Additional file 2

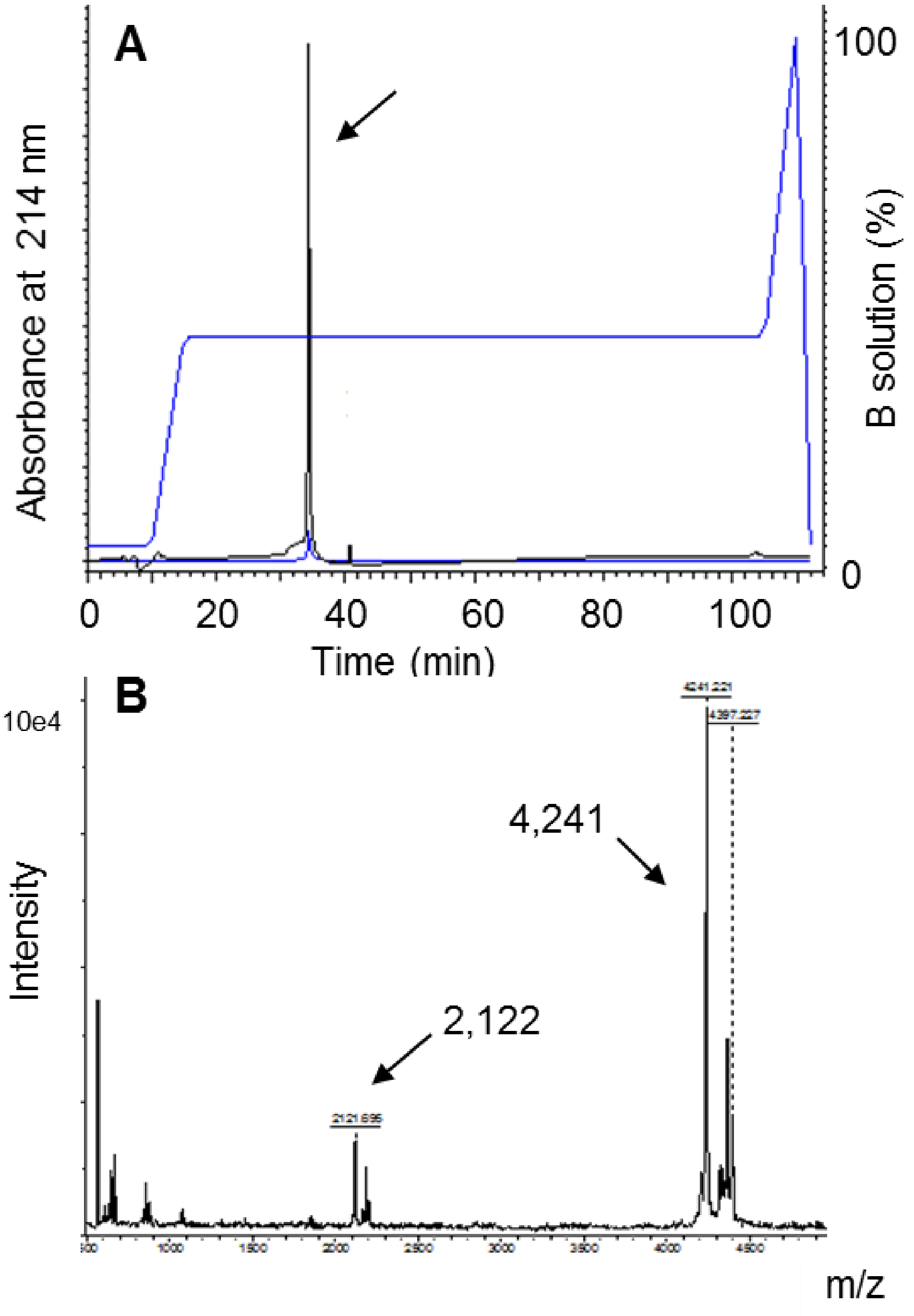

Isolation of the peptide fraction P1-RPC18 by reverse-phase rechromatography in a C18-column (RPreC-C18): (A) RPrecC-C18 profile using the same C18-column under isocratic elution condition. **(B)** Mass spectrometry (MS1) profile of the higher peak (arrow), showing the mono-charged (4,241 Da) and the doubly charged (2,122 Da) peptide ions.
