# Supplementary material for: Computer aided identification of a Hevein-like antimicrobial peptide of bell pepper leaves for biotechnological use
Source: BMC Genomics. 2016 Dec 15;17(Suppl 12):999. doi: 10.1186/s12864-016-3332-8 (PMC5249031; doi:10.1186/s12864-016-3332-8)
Supplement: Additional file 3: — Determination of the number of disulfide bonds in HEV-CANN by mass spectrometry analysis of the fraction P4-MEC after reduction and alkylation, and after alkylation only. (PDF 49 kb) [file 12864_2016_3332_MOESM3_ESM.pdf]

Additional file 3

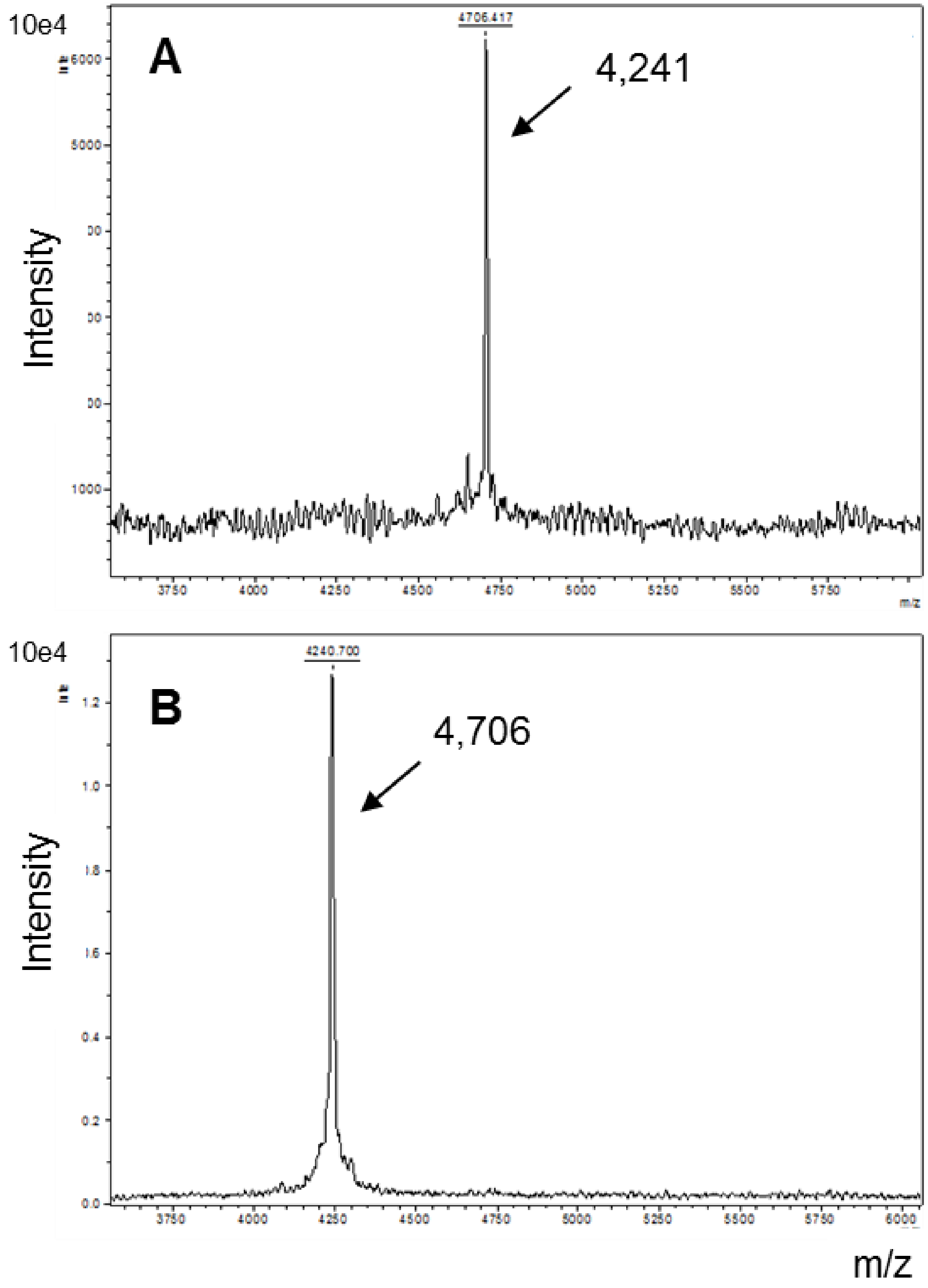

Determination of the number of disulfide bonds in HEV-CANN. (A) Mass spectrometry (MS1) profile of the fraction P4-MEC after reduction and alkylation; **(B)** MS1 profile of the fraction P4-MEC after alkylation only. Each addition of 57.05 Da indicates alkylating a sulfhydryl radical in the cysteine residue.
