# Supplementary material for: Computer aided identification of a Hevein-like antimicrobial peptide of bell pepper leaves for biotechnological use
Source: BMC Genomics. 2016 Dec 15;17(Suppl 12):999. doi: 10.1186/s12864-016-3332-8 (PMC5249031; doi:10.1186/s12864-016-3332-8)
Supplement: Additional file 4: — Alignment of the HEV-CANN sequence with PR-4 proteins from plants showing the chitin-binding domain region of the peptide Hevein from Hevea brasiliensis (residues 18 to 60) and five sequences of pathogenesis-related proteins Class-4 (PR-4). (PDF 130 kb) [file 12864_2016_3332_MOESM4_ESM.pdf]

Additional file 4

```
HEV-CANN|Hevein-UFV                ------------------------------QNCGRQAGGRVCANRLCCSQ
HEVE-HEVBR-18-60-SP                ------------------------------EQCGRQAGGKLCPNNLCCSQ
gi|590615401|ref|XP_007023212.1|   MKNTQKMGNLSLC---LVFLVSLLASTATAQQCGRQAAGRTCANNLCCSQ
gi|590642606|ref|XP_007030564.1|   ------MDKVNTVSRLLVFLVSLV-GAAVAEQCGWQAGGTICPDNLCCSQ
gi|61660944|gb|AAX51197.1|         ------MGNFGVCFLVVLTLGCLI-ATTIAEQCGRQAGGKTCPNNLCCSQ
gi|657380413|gb|KEH24556.1|        ------MGKLA-----VLILVCLI-AATIAEQCGRQAGGKTCPNNLCCSQ
gi|787035412|dbj|BAR13255.1|       -------------------------AGAVSQQCGSQAGGQLCPNNLCCSQ
                                                                 ::** **.*  *.:.*****

HEV-CANN|Hevein-UFV                FGFCGTTREYCGA--GCQSNCR----------------------------
HEVE-HEVBR-18-60-SP                WGWCGSTDEYCSPDHNCQSNCK----------------------------
gi|590615401|ref|XP_007023212.1|   FGYCGTTNEYCSPSKSCQSNCWPSG------GGGGGESASNVRATYHFYN
gi|590642606|ref|XP_007030564.1|   YGWCGNTDAYCLPENNCQSNCKSS--------GPGGETA-TVTSTYHFYN
gi|61660944|gb|AAX51197.1|         YGYCGNTDDYCSPSKNCQSNCQGGGGGG-GGGGGGGESASNVRSTYHYYR
gi|657380413|gb|KEH24556.1|        YGYCGTTDEYCGP--NCQSNCHGS--------SGGGESASNVRATYHYYQ
gi|787035412|dbj|BAR13255.1|       YGYCGSTDAYCSTANHCQSNCHGGGNTPPSPSSDIGEGANNVRATYHLYN
                                   :*:**.*  **  .  *****

HEV-CANN|Hevein-UFV                --------------------------------------------------
HEVE-HEVBR-18-60-SP                --------------------------------------------------
gi|590615401|ref|XP_007023212.1|   PPQNGWDLNAVSAYCSTWDANKPLAWRQKYGWTAFCGPVGPRGQASCGRC
gi|590642606|ref|XP_007030564.1|   PEQHGWDLMAVSAYCSTWDASKPFSWRSKYGWTAFCGPVGPTFPAACGRC
gi|61660944|gb|AAX51197.1|         PETVGGT-------------------------------------------
gi|657380413|gb|KEH24556.1|        PDQHGWDLNAVSAYCSTWDASKPYSWRSKYGWTAFCGPVGPRGQASCGKC
gi|787035412|dbj|BAR13255.1|       PQDNGWSLYAVSAYCSTWDGDQSYAWRSKYGWTAFCGPVGPHGQAACGQC

HEV-CANN|Hevein-UFV                --------------------------------------------------
HEVE-HEVBR-18-60-SP                --------------------------------------------------
gi|590615401|ref|XP_007023212.1|   LRVTNRGTRAEAIVRIVDQCSNGGLDLDAAVFQQIDTDGRGYAQGHLMVD
gi|590642606|ref|XP_007030564.1|   LRVTNTRTNAQEIVRIVDRCSNGGLDLDVGVFNRLDTDGVGYAQGHLTVK
gi|61660944|gb|AAX51197.1|         --------------------------------------------------
gi|657380413|gb|KEH24556.1|        LRVTNSGTGAQETVRIVDQCSNGGLDLDVGVFNRIDTDGRGYQQGHLIVS
gi|787035412|dbj|BAR13255.1|       LLVTNTATGAQATVRIVDQCSNGGLDLDVNVFNAIDTNGQGYAQGHLTVN

HEV-CANN|Hevein-UFV                --------------------------
HEVE-HEVBR-18-60-SP                -------------------------D
gi|590615401|ref|XP_007023212.1|   YQFVNC------------------GD
gi|590642606|ref|XP_007030564.1|   YEFVNCGDG---FNPLLSSVVDDSSK
gi|61660944|gb|AAX51197.1|         ------------------------KR
gi|657380413|gb|KEH24556.1|        YQFVDCGNELDITNPLFS--IIDAKQ
gi|787035412|dbj|BAR13255.1|       YQFVNCG------------------D
```

Alignment of the HEV-CANN sequence with PR-4 proteins from plants. The aligned sequences corresponded to the chitin-binding domain region of the peptide Hevein from *Hevea brasiliensis* (residues 18 to 60) and five sequences of pathogenesis-related proteins Class-4 (PR-4).
